# Pancreatic Cystic Lesions and Endoscopic Ultrasound Diagnostic Equipment: A Literature Review

**DOI:** 10.3390/jcm15051765

**Published:** 2026-02-26

**Authors:** Marcantonio Gesualdo, Francesco Savino, Marco Pedote, Vito Affatato, Fabio Castellano, Andrea Iannone, Martino Mezzapesa, Antonella Contaldo, Giuseppe Losurdo, Mariabeatrice Principi

**Affiliations:** Section of Gastroenterology, Department of Precision and Regenerative Medicine and Jonian Area, University of Bari, 70124 Bari, Italy

**Keywords:** pancreatic cystic lesions, endoscopic ultrasound, fine-needle aspiration, through-the-needle biopsy, confocal laser endomicroscopy

## Abstract

Pancreatic cystic lesions (PCLs) include clinically challenging conditions that range from benign to malignant prognoses. Their prevalence is increasing, and they are often detected as incidental findings during cross-sectional imaging. Thus, endoscopic ultrasound (EUS) plays a pivotal role in investigating these lesions. In this review, we analyze the complete diagnostic potential of EUS. Contrast-enhanced EUS, contrast-harmonic EUS, and elastography are useful for distinguishing between benign and malignant forms, and detective flow imaging EUS and e-FLOW EUS have enhanced the diagnostic arsenal available. Fine-needle aspiration (FNA) is important for obtaining cystic fluid for biochemical analysis and cytological examinations. Confocal laser endomicroscopy and through-the-needle biopsy represent adjunctive techniques for refined and difficult diagnosis. Moreover, artificial intelligence could be a promising modality in the EUS world. EUS allows PCLs to be detected accurately and plays a relevant role in identifying malignant forms.

## 1. Introduction

Pancreatic cystic lesions (PCLs) represent an important entity, and their prevalence is growing, likely due to incidental discoveries during cross-sectional imaging.

**Figure 1:**
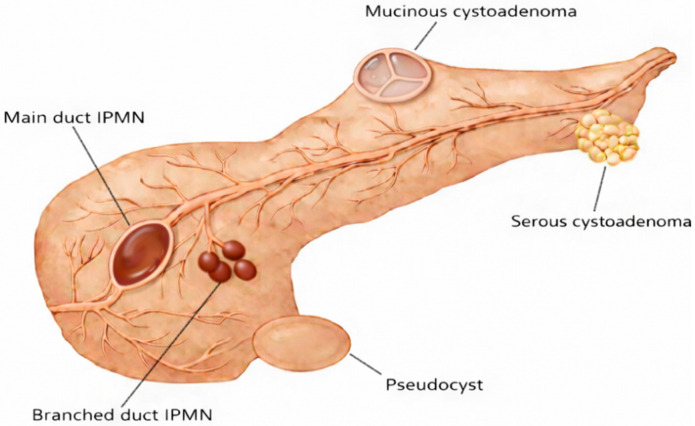
Graphical schema of the most important PCLs.

PCLs enclose cystic lesions with different characteristics and biological behaviors. The most common ones are intraductal papillary mucinous neoplasms (IPMNs), including those of the main duct (MD-IPMN) and the branched duct (BD-IPMN); mucinous cystadenomas (MCNs); serous cystadenomas (SCAs); and pseudocysts, while cystic NETs and solid pseudopapillary neoplasms (SPNs) are less common. Figure 1 graphically showcases the main features of these PCLs, and they range from benign forms (pseudocysts and SCAs) to more serious forms (MD-IPMNs, BD-IPMNs with a cyst larger than 3 cm, and MCNs), which require strict follow-up and/or surgical evaluation based on cross-sectional imaging metrics and the patient’s age.

Contrast-enhanced magnetic resonance imaging (MRI) combined with MR cholangiopancreatography (MRCP) is more sensitive than computed tomography (CT) scanning in identifying communications between PCLs and the main pancreatic duct (MPD) and determining the presence of mural nodules or inner enhanced septs [1].

At present, ERCP is rarely indicated for the evaluation of PCLs. “Fish-mouth papilla” is a pathognomonic sign of IPMN-MD, but it is only observed in 20% of patients with this disease [2]. However, ERCP with pancreatoscopy could be used to obtain IPMN-MD samples in selected cases.

In this scenario, endoscopic ultrasound (EUS) is recommended as an adjunct and relevant diagnostic technique in both European [3] and in international guidelines [4,5,6], as it is especially useful for confirming mural nodule presence and septa thickening and enhancement; sampling cyst fluid; studying indeterminate cysts after cross-sectional imaging; and detecting high-grade dysplasia/malignant forms [5,6].

In this review, we analyze the available literature on the role of EUS in PCLs.

## 2. Methods

A narrative review was planned, and a literature search was performed in the PubMed, Scopus, Embase and Cochrane Library databases. The search included papers published from 1 January 1995 up to 1 August 2025, and only English-language studies were analyzed. All authors participated in the search process and in the critical analysis of the selected publications. The following keywords were used: “Endoscopic Ultrasound; pancreatic cystic lesions; contrast enhanced-EUS; elastography; fine needle aspiration; fine needle biopsy; cytology; molecular markers; through the needle biopsy; confocal laser endomicroscopy; artificial intelligence”.

## 3. Conventional Diagnostic Techniques for PCLs

### 3.1. Conventional B-Mode and EUS Morphology

EUS is the primary method for studying unclear images of PCLs or those not fully assessed by MRI, particularly when they exhibit possibly worrisome features and high-risk stigmata [4]. This technique combines endoscopy and ultrasound to generate detailed images of the digestive tract and surrounding organs. EUS is now recommended by all guidelines for preliminary PCL evaluation, that is, mostly when there is a high risk of malignant lesions [7]. This technique has several advantages. Firstly, it provides detailed and high-resolution images with higher accuracy compared to CT and MRI, allowing for a more precise evaluation and identification of worrisome features. EUS is rated to have a sensitivity of 88%, a specificity of 53%, and a diagnostic accuracy of 70.4% for neoplastic PCLs, with better accuracy for multifocal lesion detection when compared with CT (47% versus 13%, *p* < 0.0001) or MRI (58% versus 34%, *p* < 0.0002) [8]. Additionally, it enables cytological or biopsy sampling and is helpful for a more comprehensive diagnostic approach. However, it also has some drawbacks: it is invasive, requiring sedation, and carries some procedural risks, such as infection, bleeding, pancreatitis, and, in rare cases, perforation. Furthermore, it is operator-dependent and requires extensive training to perform high-quality procedures. Every PCL has distinct EUS morphology features that could be useful for its diagnosis, as illustrated in Table 1. In most cases, SCA displays a typical microcystic morphology with a honeycomb pattern and a central scar, while MCN is unilocular with typical eggshell calcifications [7]. In contrast, BD-IPMN is connected to the main pancreatic duct and has a typical “bunch-of-grapes aspect”.

### 3.2. Contrast-Enhanced EUS and Contrast-Enhanced Harmonic EUS

Contrast-enhanced endoscopic ultrasonography (CE-EUS) is an important tool in the EUS armamentarium. It allows real-time evaluation following the intravenous administration of a contrast agent. Contrast-enhanced harmonic EUS (CH-EUS) is an adjunctive modality used to assess contrast-enhanced EUS features, as it has the ability to reduce artifacts. These tools are effective for distinguishing between malignant and benign pancreatic masses. CH-EUS utilizes a contrast agent combined with tissue harmonic imaging technology to differentiate blood flow characteristics in PCLs, making it particularly useful for the characterization of malignant mural nodules, as well as the septa [9]. The contrast agents used include microbubbles encased in a lipid shell, enhancing real-time visualization and, therefore, allowing precise microvascularization assessments during ultrasound (US) imaging. Real-time EUS visualization can be achieved through either the color Doppler mode or with a dedicated second-harmonic contrast mode. These contrast agents highlight different enhancement phases, including the arterial phase (10–30 s after administration) and the venous phase (30–120 s). The main role of CH-EUS is to provide information on tissue microvascularization, thereby aiding in the differential diagnosis between enhanced mural nodules and other non-enhanced solid parts. Thus, CH-EUS is included in the diagnostic algorithms for pancreatic lesions (PLCs) in both international and European guidelines [3,4,6]. The authors of a 2021 meta-analysis reported that CH-EUS has good sensitivity (88.2%) and relatively high specificity (79.1%) for diagnosing mural nodules with high-grade dysplasia (HGD) or invasive carcinoma, and its sensitivity further improved (reaching 90%) in studies using contrast harmonic mode compared to those using color Doppler only [10]. An important advantage of this technique is its ability to enhance the sensitivity of EUS-guided fine-needle aspiration (EUS-FNA) when combined with CH-EUS. CH-EUS helps avoid the puncture of necrotic areas (which do not enhance) and inflammatory zones (which show iso- or hyper-enhancement), which may be located at the center or around the malignant mass [11,12]. The only downsides of using CH-EUS are allergic reaction risks and known right-to-left pulmonary shunts or relevant pulmonary hypertension (pulmonary artery pressure more than 90 mmHg) [12].

### 3.3. EUS Elastography

Elastography has traditionally been considered challenging for pancreatic evaluation due to its limited accuracy and reproducibility. This is mainly because the pancreas is a small organ located deep within the human body. However, this belief is evolving with the advent of EUS elastography (EUS-E), which provides a useful, non-invasive tool for predicting the nature of pancreatic lesions. EUS-E is performed using a conventional ultrasonographic endoscope and requires no additional instruments. It is typically conducted with a two-panel image: one side shows the conventional B-mode EUS image, while the other side displays the elastography picture, which provides information on tissue stiffness. This is facilitated by a color scale corresponding to the degrees of stiffness of the tissue, from red (soft tissue) to blue (solid lesion), thus helping the operator understand the tissue’s potential nature.

Pancreas elastography can be performed using two modalities:

(a) Strain Elastography: This technique is based on the principle that certain pathologies, including cancer, induce changes in the stiffness of the affected organ [13] and on small deformations caused by compression in B-mode, followed by software analysis of the deformation degree. The analysis is performed in real time and produces a color-coded stiffness map, where harder tissues, moderately hard tissues, intermediate tissues, medium-soft tissues, and soft tissues are represented in dark blue, cyan, green, yellow, and red, respectively [14]. This evaluation can be further improved with the use of the strain ratio, as it allows the operator to select two regions of interest (ROIs) [14].
(b) Shear Wave Elastography: It is a non-invasive method used to assess tissue stiffness and elasticity. This technique measures the speed of waves generated by acoustic radiation force within the tissue, as the speed of wave propagation is directly related to tissue elasticity [15].

EUS-E is particularly useful for detecting solid pancreatic lesions [16,17], but it also plays a key role in PCLs with solid components, such as in an IPMN with a mural nodule. Indeed, mural nodules show contrast uptake, while a mucin clot does not. EUS elastography offers several advantages: real-time assessment, with no additional tools required; minimal additional risks or costs; only a slight increase in examination duration; no specific patient preparation is necessary; a rapid learning curve, requiring minimal training; excellent interobserver agreement; and an extremely high negative predictive value for malignancy, making it especially useful for small pancreatic tumors and for determining when and where to perform fine-needle aspiration (FNA) or in the evaluation of cases where FNA cannot be performed [14,18,19]. Despite its advantages, EUS elastography has some limitations. Its main disadvantage is the inability to control tissue compression caused by the EUS transducer. Furthermore, it becomes less reliable when large masses are present, particularly when insufficient surrounding tissue is available for accurate ROI analysis. Many authors are reluctant to use this technique due to a lack of sufficient data demonstrating its clinical benefit compared to conventional EUS, with or without EUS-guided tissue acquisition. Additionally, studies evaluating elastography often overestimate its accuracy, as they typically do not include lesions of an indeterminate nature, which may not fully reflect the real-world diagnostic challenges of the technique [20,21].

### 3.4. Detective Flow Imaging and E-FLOW EUS

Since the introduction of CH-EUS, researchers have sought faster, more cost-effective, and safer methods to analyze PCL microvascularization. Initially, Doppler ultrasound was employed, but it was later surpassed by eFLOW, a high sensitivity Doppler technique that became the most used tool for blood flow assessment in EUS [22]. Despite its effectiveness, eFLOW has limitations in detecting low-velocity or microvascular flow patterns. To address this, detective flow imaging (DFI-EUS) was developed [23]. DFI-EUS is an advanced imaging technique that provides highly dynamic observation of low-velocity blood flow, which is below the detection threshold of conventional color Doppler methods, with a high frame rate. Similarly to Doppler imaging, DFI-EUS can be used without contrast agents, offering greater sensitivity in detecting microcirculation compared to traditional Doppler imaging techniques. Due to its non-invasive nature, high sensitivity, and simplicity, DFI-EUS has the potential to replace CE-EUS when evaluating microvascular blood flow. However, studies investigating its effectiveness are limited, and its diagnostic accuracy for IPMNs has not been fully validated so far [24]. One of the most significant studies in this field is the one by Endo K. et al. [25]. In their retrospective analysis, 68 patients with mural nodules within IPMNs were evaluated blindly by three expert endosonographers, where they reviewed CE-EUS and DFI-EUS without access to patients’ clinical information. CE-EUS demonstrated contrast enhancement of mural nodules in 24 cases. Among these, DFI-EUS detected blood flow signals in 20 patients, with no false-positive results reported. DFI-EUS achieved a sensitivity of 83%, a specificity of 100%, and an overall accuracy of 93% in detecting blood flow within mural nodules. Interobserver agreement was substantial, with kappa values ranging from 0.6 to 0.8 [25]. In another study, Yamashita Y. et al. enrolled 54 patients with solid pancreatic lesions, IPMNs, or enlarged lymph nodes, which were analyzed using CE-EUS, eFLOW-EUS, and DFI-EUS. The vessel detection capability of DFI-EUS was assessed across 53 lesions. Compared with CE-EUS, DFI-EUS showed superior vessel assessment rates: a sensitivity of 91%, a specificity of 100%, and an accuracy of 92%. In contrast, eFLOW-EUS showed a sensitivity of 53%, a specificity of 100%, and an accuracy of 60%. These results suggest that DFI-EUS is significantly more effective than eFLOW-EUS for vessel detection, likely due to its enhanced visualization of fine vasculature compared to conventional Doppler techniques. Nevertheless, DFI-EUS failed to detect vessels in four out of 46 lesions (9%), while CE-EUS successfully identified vascular signals; specifically, it demonstrated lower detection rates in pancreatic cancer cases [24].

### 3.5. EUS-Guided Sampling

**Figure 2:**
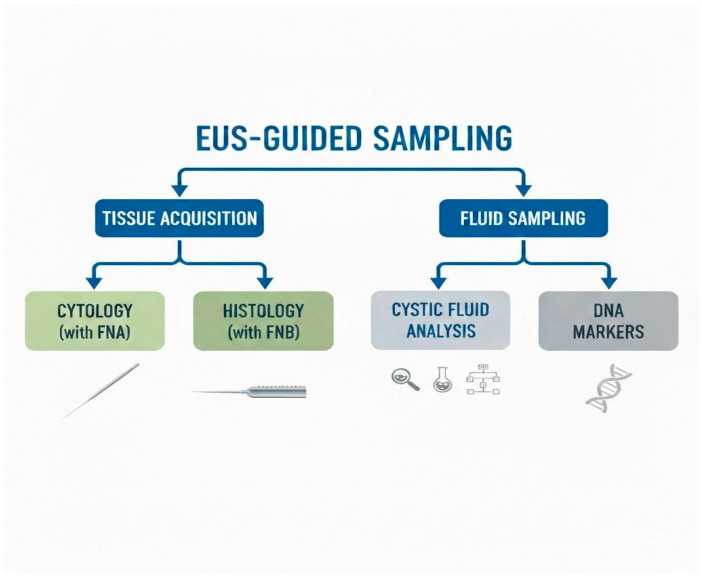
EUS-guided sampling modalities in PCLs.

EUS is recommended following the identification of a lesion via cross-imaging modalities or when there is significant clinical suspicion. In recent years, the term EUS-FNA has been largely considered synonymous with PCL-guided sampling. However, in a recent Japanese paper, Isayama et al. [26] proposed “EUS-guided sampling” as the correct term; indeed, this term comprises both cytological–histological sampling (EUS tissue acquisition, EUS-TA) and EUS-guided fluid sampling used for biochemical analysis and for detecting molecular markers (Figure 2).

#### 3.5.1. Tissue Acquisition

EUS-TA involves puncturing the target organ in the gastro-intestinal (GI) system using a real-time EUS navigation system to obtain the required pathology samples for histopathological and/or cytological analysis and encompasses both EUS-guided fine-needle aspiration (EUS-FNA) and EUS-guided fine-needle biopsy (EUS-FNB) [27]. The selection of a specific EUS-TA approach is influenced by three fundamental factors: (a) the type of lesion (cystic or solid), (b) the endoscopist’s preference, and (c) available resources at disposal [27]. EUS is advocated as a supplementary tool to various imaging techniques in all guidelines, and it can be subsequently utilized for identifying invasion, where it proves to be most efficient in outlining malignant features [3]. Over the years, different types of needles have been developed for EUS-FNA and FNB, each with specific characteristics that affect sample quality and procedural safety. Thus, FNA needles with different features and sizes are available, and the main types are as follows [27]:

(a) Lancet needle tips with sizes of 19G, 22G, and 25G: They are easily introduced and safe but may occasionally result in insufficient tissue acquisition and blood contamination.
(b) Menghini needle tips that are flexible for easier insertion or echogenic tips with usual diameters of 22G or 25G: They are easy to maneuver and have a lower risk of complications. However, they are limited by inadequate material for advanced cytology studies.

EUS-FNA needles are useful for cystic lesions, but the main problem is their inability to obtain histological core tissue in lesions with solid components or in mural nodules in PCLs. Moreover, their diagnostic efficacy relies on a cytopathologist providing a rapid on-site evaluation (ROSE), and they may provide insufficient tissue for risk stratification and customized anti-cancer treatment. Taken together, these factors shifted the focus of endoscopists toward the development of “EUS-biopsy” needles to overcome the limitations of FNA needles, and in recent years, several FNB needles that are designed to optimize tissue harvesting and better preserve histologic architecture have been developed. The main types include the following [27]:

(a) Franseen needles: They are characterized by a triangular, three-edged tip, which facilitates penetration and retrieval of well-preserved tissue cylinders, making them excellent for pancreatic and lymph node biopsies due to a maximization of EUS-TA and a reduction in sample destruction.
(b) Fork-tip needles: They have a fork tip with six cutting-edge surfaces (“shark” form), making them ideal for obtaining samples for histological analysis, as well as molecular and immunohistochemical analyses.

Each needle has specific advantages and indications, and the choice often depends on both the clinical question and the dexterity of the endoscopist; however, the type of lesion (cystic or solid) is crucial. EUS has now been accepted as an important modality to evaluate pancreatic cysts due to the following reasons [28]: its ability to identify findings suggestive of high-grade dysplasia/invasive carcinoma (high-risk stigmata); its ability to confirm the presence of a mural nodule; and that it is capable of sampling cyst fluid or solid components, as well as indeterminate cysts after MRI/CT. The discussion regarding EUS-FNA compared to EUS-FNB has been contentious since the introduction of the FNB needle in 2002. As molecular testing progressed, the fundamental drawbacks of EUS-FNA needles, particularly their inability to yield a histological tissue core, have become increasingly evident. The notion of EUS-FNA as the ‘gold standard’ faced scrutiny with the emergence of direct comparisons to FNB needles, as EUS sampling could only be performed if it could change treatment options or in doubtful cases, as described in both European [3] and American guidelines [29]. In the “PCL world”, tissue acquisition could be obtained with FNA or FNB when solid components or mural nodules are present. Cytologic or histological samples allow us to differentiate between benign and malignant lesions and perform differential diagnosis between PCLs and other lesions [28]. Cyto-histology is useful for studying atypical cells/dysplasia, and the main cyto-histopathological aspects to be evaluated in this pathology report are as follows:

(a) Cellularity, which is often limited: The presence of mucinous epithelial cells suggests mucinous cysts (IPMNs or MCNs).
(b) Atypical/neoplastic cells, which indicate suspicion of malignancy.
(c) Mucin, where the presence of extracellular mucin is a key marker for mucinous cysts.
(d) Serous cells, which are typical cuboidal cells of SCA and usually non-mucinous.

Cytology has high specificity but poor sensitivity, and the limitation of cytological evaluation is due to its relatively low sensitivity despite being highly specific, resulting in inadequate diagnostic yield in a considerable number of cases, reported to be over 50%. [30]. As noted by several authors who performed meta-analyses [31,32] and reiterated by many guidelines, [4,5], this limitation mainly depends on the number of cells contained in the cystic fluid. Thus, mucus analysis on the intra-cystic fluid aspirate should always be performed. Although mucin may be macroscopically visible during aspiration, it is necessary to identify a “thick” layer of colloidal mucin that covers much of the microscope slides. Therefore, it is recommended to perform the smear in the endoscopy room or to send the material to the laboratory, thus avoiding fixatives that might compromise mucus identification. This may be helpful for the diagnosis of a mucinous cyst, even if it is acellular [33,34]. Moreover, EUS is very important in the follow-up of PCL patients with a high risk of malignancy, as it allows us to perform FNA/FNB when there is suspicion; indeed, in a lot of cases, pancreatic carcinoma PCLs are derived from mural nodules or thickened walls [35]. At the same time, EUS is very important for the detection of pancreatic adenocarcinoma (PDAC), both concomitant with and independent of PCL presence, as PCL patients occasionally develop a primitive PDAC regardless of a cystic lesion [36].

#### 3.5.2. Fluid Sampling and Cystic Fluid Analysis

Fluid sampling is a part of EUS sampling, as described before [26], and cystic fluid analysis is a very useful tool in the EUS armamentarium as it allows us to assess PCLs. Firstly, the “string sign” is an empirical technique to differentiate between mucinous and non-mucinous PCLs, and it consists of placing a drop of cyst fluid aspirate between the thumb and index finger and stretching it; a string length > 3.5 mm is suggestive for a mucinous PCL [3]. Several biochemical investigations on cystic fluid are available to help identify the type of lesion and its potential evolution, and some studies [4,5] demonstrated that the string sign is highly accurate for diagnosing pancreatic mucinous cystic neoplasms and may be used as an important tool to support diagnostic accuracy (sensitivity: 93.8%; specificity: 85.7%; accuracy rate: 92.3%). The most important and commonly used biochemical tests are the carcinoembryonic antigen (CEA), amylase, and glucose assays (Figure 2).

(a) CEA: Elevated intracystic CEA levels may allow us to distinguish mucinous (IPMN or MCN) from non-mucinous cysts but not malignant from benign cysts [37]. The cut-off value varies from 20 ng/mL to 800 ng/mL in different studies, with higher sensitivity for low cut-offs and higher specificity for higher cut-offs. However, the most frequently used cut-off comes from a prospective study by Brugge et al. [38] on 112 patients undergoing surgery. Their study determined that a level ≥ 192 ng/mL had a diagnostic sensitivity of 75%, a specificity of 84%, and an accuracy of 79% in differentiating between mucinous and non-mucinous cysts. However, CEA values significantly differ according to individual assays, with various optimal cut-offs reported in the literature for non-mucinous cysts. A recent comparative study [39] proposed a strategy to identify differences in thresholds by analyzing the optimal CEA cut-off value for pancreatic cysts from two different tests (Beckman Dxl (BD) or Siemens Centaur XP (SC)). The optimal CEA cut-off value for all samples at the study’s institution was 45.9 ng/mL [area under the curve (AUC) = 86, sensitivity = 85.7%, and specificity = 73.8%]. Based on the sub-analysis of the CEA assay, the cut-off values were 45.9 ng/mL (AUC = 84.27, sensitivity = 89.7%, and specificity = 71.4%) for BD and 24.4 ng/mL (AUC = 77, sensitivity = 81.8%, and specificity = 75%) for SC (*p* = 0.48) [39].
(b) Intracystic glucose assay: Glucose is also useful in the differential diagnosis between mucinous and non-mucinous lesions. A multicenter study involving 93 patients indicated that intracystic glucose was superior to CEA for mucinous cystidentification when determined at a glucose threshold of 50 ng/mL (AUC = 0.81) [40]. Low levels of intracystic glucose (<50 ng/mL) are indicative of mucinous pancreatic cysts. Moreover, combining tests did not enhance the diagnostic precision compared to the glucose test alone. Further benefits of glucose measurement include the capability for real-time analysis in the examination room using a glucometer, as well as the requirement of only a small volume of fluid. Thus, assessing intracystic glucose may hold potential diagnostic relevance for indeterminate cysts, where CEA levels range from 5 to 192 ng/mL, though this fact requires additional investigation. Considering the enhanced sensitivity and diagnostic precision of pancreatic cyst fluid glucose over CEA by itself, the authors of a meta-analysis [41] explored the efficacy of combined testing with both methods, and the findings that indicated low glucose levels or high CEA were noted in four studies (348 lesions), with a pooled sensitivity and specificity of 97% (95% CI, 90–99) and 72% (95% CI, 47–88), respectively. The diagnostic accuracy for combination testing reached 97% (95% CI). However, although the sensitivity and diagnostic accuracy of combining both the glucose and CEA tests showed substantial superiority compared to CEA alone (*p* < 0.001), combination testing did not yield better testing results than pancreatic cyst fluid glucose sampling alone.
(c) Amylase: Amylase levels in pancreatic cystic fluid are assessed to elucidate potential communication between the cyst and the pancreatic duct or secondary ducts. High amylase levels (>250 UI/L) confirm communication with the MPD (as in IPMNs and pseudocysts). MCNs very rarely exhibit macroscopic communication with the pancreatic duct, so the expected level of amylase is usually low, as observed in SCA. However, several studies [42,43,44] have shown that amylase levels in different MCNs can be elevated, with no particular differences between IPMNs and MCNs, most likely due to microcommunication between the cyst and the ductal system. A summary of cyst fluid analysis according to PCL type is reported in Table 2.

#### 3.5.3. Molecular Markers

Next-generation DNA sequencing (NGS) of pancreatic cystic lesions represents a revolution in the diagnostic and therapeutic management of such lesions, as it increases accuracy compared with cytologic and radiologic criteria alone, thereby allowing more accurate risk stratification. In particular, the presence of mutations in KRAS and GNAS suggests a mucinous neoplasm. Mutations in GNAS were only observed in IPMNs but not in MCNs, and KRAS mutations are likely suggestive of mucinous, but not necessarily malignant, cysts [45]. Mutations in VHL are associated with serous cysts and are thus benign. Maher et al. [46] demonstrated that a multiomics biomarker approach with elevated pancreatic cyst fluid (PCF) miRNAs with mutant KRAS, mutant GNAS, and serum CA19-9 may be useful to detect high-risk cysts for early clinical intervention. TP53, PIK3CA, and PTEN are molecular prognostic markers, and mutations in these genes are associated with more advanced stages of disease. In 2023, Paniccia et al. proposed a prospective, multicenter study on the use of real-time next-generation sequencing (NGS) of pancreatic cystic fluid to identify genomic alterations that are useful for the clinical management of pancreatic cysts [47]. The study involved more than 1600 patients and demonstrated that NGS of cystic fluid, obtained by EUS-FNA, allows more accurate cyst-type classification than traditional methods such as cytology, tumor markers, and imaging. It also highlighted how TP53 mutations are strongly associated with advanced neoplasia (high-grade dysplasia and carcinoma) in pancreatic mucinous cysts, particularly IPMNs. The presence of TP53 had high specificity (97–99%) but low sensitivity (15–18%) for advanced lesions. The combination of TP53 with other mutations, such as SMAD4, PTEN, and PIK3CA, increased the predictive value for advanced neoplasia. PTEN alterations (mutations or loss of expression) were also markedly associated with advanced lesions [47]. As with TP53, the presence of PTEN indicated a high risk of malignant progression and supported more aggressive clinical decisions (surgery or close follow-up) [47]. Additionally, for PTEN, specificity was very high (98–99%), but sensitivity remained limited (10–13%). PIK3CA mutations were rare but highly specific for IPMNs with high-grade dysplasia or carcinoma [47]. The article pointed out that the presence of PIK3CA, especially in association with TP53 and PTEN, was a marker of advanced malignancy, as indicated by low sensitivity (3–5%) but very high specificity (100%) [47]. Additionally, molecular markers are also useful in doubtful cases; for example, a KRAS mutation is a hallmark of a neoplasia-derived degenerated IPMN, as opposed to a primitive PDAC that does not express this mutation [36].

## 4. Ancillary Techniques for PCNs

### 4.1. Through-the-Needle Biopsy (TTNB)

EUS through-the-needle biopsy (TTNB) has emerged as a promising tool for overcoming the diagnostic limitations of EUS-FNA in pancreatic cystic lesions (PCLs), particularly for accurate cyst-type classification and neoplastic risk stratification. Several meta-analyses have confirmed its superior diagnostic performance over FNA [48], especially in mucinous cysts with nodules [49,50]. The technique employs microforceps (e.g., Moray™ or Micro Bite™) passed through a 19G FNA needle, enabling tissue acquisition from the cyst wall or mural nodules for histologic analysis [51,52]. The microforceps typically have a diameter of 1 mm and easily pass through 19G needles. Although the procedure lacks standardization, key technical factors include the sequence of fluid aspiration (typically after TTNB) [53,54], the use of preloaded forceps [55], and obtaining at least two visible specimens [48]. Indications for EUS-TTNB include indeterminate PCLs on imaging or FNA [31,56,57,58], cyst subtyping (e.g., SCA, MCN, IPMN, SPN, or cNET) [59], risk stratification in IPMNs [60], and molecular profiling through next-generation sequencing (NGS) [61,62], while contraindications include clearly benign lesions, an expected low clinical impact of histology, small or inaccessible cysts, and high-risk patients [63,64]. Additionally, safety remains a concern, with adverse events (AEs) reported in 8.6–10.1% of cases [65,66]. Specifically, intracystic bleeding and pancreatitis are most frequent, and mild prophylactic strategies, such as rectal NSAIDs and hydration, often have no clear benefit [67,68]; however, antibiotic prophylaxis may be useful when cyst aspiration is incomplete [67,68]. EUS-TTNB significantly enhances diagnostic yield across cyst types and improves clinical decision-making, modifying management in up to 39% of cases [69]. Compared to molecular fluid analysis and needle-based confocal laser endomicroscopy (CLE), TTNB shows comparable diagnostic accuracy and safety, with particular strength in detecting malignant lesions [70,71].

### 4.2. Confocal Laser Endomicroscopy (CLE-Cellvizio)

CLE is an advanced endoscopic method that enables real-time imaging at a subcellular resolution, providing an “optical biopsy”. This technique holds the potential to significantly improve the diagnostic accuracy of EUS-FNA for both PCLs and solid pancreatic masses (SPMs) [72]. CLE employs a probe-based CLE (pCLE) system (e.g., Cellvizio Endomicroscopy System, Mauna Kea Technologies), where a flexible fiber bundle transits through the working channel of a standard endoscope or through a 19-gauge EUS-FNA needle (needle-based CLE, nCLE) [73], and a prototype for 22-gauge puncture needles has also been developed [74]. For imaging, an intravenous fluorescent agent (typically 2.5 mL of a 10% fluorescein sodium solution) is given 30 s to 3 min before nCLE imaging, and its fluorescence is optimally captured from a few seconds up to 8 min post-injection [75]. Fluorescein is a non-toxic, FDA-cleared agent that highlights the extracellular matrix and vasculature. The nCLE procedure generally takes approximately 6 min to complete and is particularly effective in characterizing mucinous cysts (e.g., IPMNs and MCNs), achieving a sensitivity of 94.9% and an accuracy of 91.4% [1]. These characteristic features include papillary or villous epithelial projections, epithelial bands, and occasionally large-caliber vessels or dark cell clusters [76]. For serous cystadenomas (SCAs), the “fern pattern” or superficial vascular network is considered pathognomonic, with reported sensitivities of 95–99% and specificity approaching 100% [77]. In contrast, pseudocysts are identified by their acellular content, as they lack epithelial structures [6], while cystic neuroendocrine tumors (NETs) display compact cell clusters, fibrotic stroma, and trabecular patterns [78]. Compared to standard diagnostic modalities, nCLE outperforms EUS-FNA (OR 3.94; *p* = 0.003) and CEA-based fluid analysis, both of which tend to show <50% diagnostic accuracy [1]. nCLE also demonstrates higher AUROC than CEA and EUS morphology in distinguishing mucinous from non-mucinous PCLs and benign from premalignant lesions [6]. Combining both EUS-TTNB and nCLE significantly improves diagnostic performance, with sensitivity and the NPV reaching 100% and an AUROC of 0.947 [79]. Despite being a promising technique, nCLE has some limitations: its availability is restricted due to high costs, limited operator experience, and its niche role outside clinical trials [77], and technical challenges include a steep learning curve, difficulty in maintaining stable imaging, and a narrow field of view—visualizing only ~30% of the epithelium [78]. Interobserver agreement varies, especially among less experienced users. Additionally, most data are derived from high-volume centers, and the lack of large RCTs limits generalizability. Lastly, despite the procedure being well tolerated, adverse events such as mild pancreatitis (1.3–6.6%) and bleeding have been reported [75,76].

### 4.3. Artificial Intelligence

Integrating artificial intelligence (AI) into EUS could be a significant step in the diagnosis and management of pancreatic diseases. Initially, AI applications relied on computer-aided detection (CADe) and diagnosis (CADx) systems to identify and classify abnormalities within EUS images. Over time, these methods have evolved into more sophisticated machine learning (ML) and deep learning (DL) models, particularly convolutional neural networks (CNNs), which have demonstrated exceptional performance in medical image interpretation and classification [80,81,82]. Beyond EUS, AI has shown diagnostic promise in pancreatic radiomics and digital pathology. DL algorithms applied to CT imaging and MRI have enabled earlier detection of pancreatic neoplasms such as IPMN and pancreatic intraepithelial neoplasia (PanIN), as well as the prediction of underlying somatic mutations [83,84]. Similarly, AI-based digital histopathology has accelerated diagnostic workflows and improved accuracy, thereby facilitating early identification of malignant progression in premalignant lesions [85]. Within EUS, AI has been applied both as a diagnostic enhancer and as a training aid. For example, the BP MASTER system—a CNN-based model developed in Wuhan—was designed to improve anatomical recognition and image segmentation during EUS procedures [86,87]. Another AI system, CH-EUS MASTER, specifically trained to analyze contrast-enhanced EUS (CH-EUS), improved lesion localization accuracy and reduced procedure times [88]. In the context of PCLs, AI has demonstrated efficacy in distinguishing benign from potentially malignant forms. Kuwahara et al. developed a CNN model capable of predicting the malignancy of IPMNs with an accuracy of 94%, significantly outperforming expert clinicians (56%) [89]. Although limited by surgical-only cohorts, these findings support the diagnostic potential of AI. Additionally, other studies focused on differentiating specific cystic subtypes, such as mucinous cystic neoplasms (MCNs) and serous cystic neoplasms (SCNs). For instance, the model by Nguon et al. achieved an accuracy of 0.80 [90], while Vila-Boas et al. reported an accuracy of 0.985 in distinguishing mucinous from non-mucinous lesions using 5505 EUS images from 28 patients [91]. Despite these encouraging developments, several limitations hinder the full clinical integration of AI into pancreatic EUS. The lack of standardized data acquisition protocols, limited multicenter datasets, and the unclear decision-making process of deep learning algorithms reduce interpretability and generalizability. Moreover, ethical concerns regarding patient data privacy and security must be addressed, particularly given the high data volumes required for AI training [92].

## 5. Conclusions

PCLs show a wide variety of behaviors. To address this, EUS morphology, cytology, cyst fluid analysis, and adjunctive techniques are used to obtain greater accuracy in studying PCLs and their malignant risk. Thus, although EUS is useful for surgical referral in cases of malignant findings, it is important to tailor decisions based on age, comorbidities, and cystic characteristics.

## Figures and Tables

**Table 1:** The main clinical, morphological, and endoscopic features of PCLs.

Cyst Type	Typical Demographics	Clinical Presentation	Location	Imaging Features	Duct Communication/MPD	Cyst Fluid/Biology [5]	Multifocality	Malignant Potential [7]
**Pseudocyst**	♂ predominance (<25% female) 4th–5th decade	History of acute or chronic pancreatitis	Variable	Unilocular or multilocular, thin wall, and homogeneous fluid	May communicate with MPD; MPD may be irregular or contain stones.	Non-mucinous, very high amylase, and low CEA	Rare	0%
**Serous cystic cystadenoma (SCA)**	♀ ~70% 6th–7th decade	Mostly asymptomatic	Any	Microcystic or mixed micro/macro-cystic, central stellate scar ± calcification, and honeycomb appearance	No communication; MPD normal or deviated	Serous fluid, very low CEA, VHL mutation, and wild-type KRAS/GNAS	~50%	~0%
**Branch-duct IPMN (BD-IPMN)**	Equal sex distribution 6th–7th decade	Often asymptomatic; may cause pancreatitis.	Head > body/tail	Cystic lesions with ductal communication, grape-like clusters, and multiplicity	Yes; MPD usually normal or mildly dilated	Mucinous fluid, low glucose, high CEA, KRAS and/or GNAS mutations	20–40%	1–38%
**Main-duct IPMN (MD-IPMN)**	Equal sex distribution 6th–7th decade	Often symptomatic	Diffuse	MPD dilation >5–10 mm, “fish-mouth” papilla, and intraductal filling defects	Yes; marked MPD dilation	Mucinous fluid, low glucose high CEA, KRAS, and/or GNAS mutations	Common	33–85%
**Mucinous cystic neoplasm (MCN)**	♀> 90% 4th–6th decade	Mostly asymptomatic	Body/tail (≈95%)	Unilocular or oligolocular, thick wall, septations, and peripheral “eggshell” calcifications	No; MPD normal or deviated	Mucinous fluid, low glucose, high CEA, KRAS mutation, and wild-type GNAS	No	10–34%
**Solid pseudopapillary neoplasm (SPN)**	♀ ~ 90% 2nd–3rd decade	Often incidental	Tail > head	Heterogeneous solid-cystic lesion, hemorrhagic components, calcifications	No	Variable; β-catenin mutation	No	10–15%
**Cystic neuroendocrine tumor (cNET)**	Variable age/sex	Mostly asymptomatic; ~10% functional	Any	Thickened enhancing wall or solid component and hypervascular	No	Low CEA; may show NET markers.	Rare	5–10%

**Table 2:** Schema summarizing the concentration of different laboratory determinations according to the type of pancreatic cyst. (a) CEA cut-off: 192 ng/mL. (b) Amylase cut-off: 250 UI/L. (c) Glucose cut-off: 50 ng/mL.

	Pseudocyst	SCA	MCN	IPMN	SPN
**Mucin**			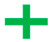	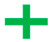	
**CEA (a)**	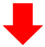	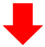	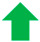	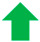	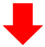
**Amylase (b)**	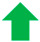	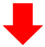	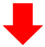	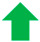	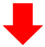
**Glucose (c)**		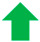	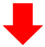	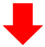	

The symbols mean respectively: positive, increased, decreased.

## Data Availability

No new data were created or analyzed in this study.
